# Comparative Clinical Value of Pharmacologic Therapies for B-Cell Chronic Lymphocytic Leukemia: An Umbrella Analysis

**DOI:** 10.3390/jcm11071868

**Published:** 2022-03-28

**Authors:** Monia Marchetti, Paolo Rivela, Claudia Bertassello, Manuela Canicattì

**Affiliations:** 1 SC Ematologia, Azienda Ospedaliera SS Antonio e Biagio e Cesare Arrigo, 15121 Alessandria, Italy; paolo.rivela@ospedale.al.it (P.R.); cbertassello@ospedale.al.it (C.B.); 2 Oncology Unit, Hospital Cardinal Massaia, ASLAT, 14100 Asti, Italy; manuela.madesse@gmail.com

**Keywords:** chronic lymphocytic leukemia, ibrutinib, acalabrutinib, venetoclax, obinutuzumab, chlorambucil, rituximab, meta-analysis, network meta-analysis

## Abstract

Several new drugs are progressively improving the life span of patients with B-cell chronic lymphocytic leukemia (CLL). However, the rapidly evolving standard of care precludes robust assessments of the incremental clinical value of further innovative drugs. Therefore, we systematically reviewed comparative evidence on newly authorized CLL drugs, as reported by standard and network meta-analyses (MA) published since 2016. Overall, 17 MAs addressed the relative survival or safety of naïve and/or refractory/relapsed (R/R) CLL patients. In R/R patients, therapies including BTK- and BCL2-inhibitors reported progression free survival (PFS) hazard ratios ranging from 0.08 to 0.24 (versus chemotherapy) and a significant advantage in overall survival (OS). In naïve patients, the PFS hazard ratios associated with four recent chemo-free therapies (obinutuzumab- and/or acalabrutinib-based) ranged from 0.11 to 0.61 versus current standard treatments (STs), without a significant OS advantage. Ten MAs addressed the risk of cardiovascular, bleeding, and infective events associated with BTK inhibitors, with some reporting a different relative safety in naïve and R/R patients. In conclusion, last-generation therapies for CLL consistently increase PFS, but not OS, and minimally decrease safety, as compared with STs. Based on available evidence, the patient-customized adoption of new therapies, rather than universal recommendations, seems desirable in CLL patients.

## 1. Introduction

B-cell chronic lymphocytic leukemia (CLL) is a rare neoplasm, accounting for 1.2% of overall new cancer diagnoses. It has an indolent course and affects 56 per 100,000 people (https://seer.cancer.gov/statfacts/html/clyl.html, accessed on 30 January 2022). Moreover, 5-year overall survival (OS) has increased to over 86% and mortality has decreased to 1.1/100,000 patients per year in the last 20 years [1].

It is estimated that the recent approval of several novel drugs and combination treatments can potentially save further years lost, despite the old age at which CLL usually occurs [2]. Frontline treatment with BCL2 inhibitors (venetoclax) or BTK inhibitors (BTKis), possibly associated with anti-CD20 monoclonal agents—especially obinutuzumab (O)—is currently being proposed as the standard of care for most CLL patients, despite their molecular risk [3]. As a consequence of the widespread availability of such treatments, mean overall survival (OS) is expected to exceed 10 years and quality-adjusted life expectancy to be higher than 7.6 quality-adjusted life years [4]. Despite the clinical improvements associated with novel drugs, CLL-related drug expenditures have already increased by almost 10-fold [5], questioning the competitive value-for-cost of CLL therapies, especially continuous ones, as compared with other healthcare interventions. Moreover, side effects might change the clinical value of new drugs that are currently widely accepted in the real world. Finally, even though technology assessments of newly proposed treatments take into account different standard-of-care treatments, randomized trials usually include few treatments.

Conducting a systematic review of evidence and meta-analyses (MA) can help in translating wide volumes of clinical research to assist in decision making. Network meta-analyses (NMA) are particularly useful MAs that allow the indirect comparison of treatments with head-to-head studies by using the Bayesian inferential analyses of several chained studies. However, as an overwhelming number of MAs and NMAs have been reported in the last years, a systematic review method for published MAs/NMAs has been developed. “Umbrella reviews” represent the highest level of evidence synthesis currently available [6].

The present umbrella review aims to examine published MAs and NMAs in order to estimate the incremental clinical value of novel versus standard therapies for CLL.

## 2. Methods

**Figure 1:**
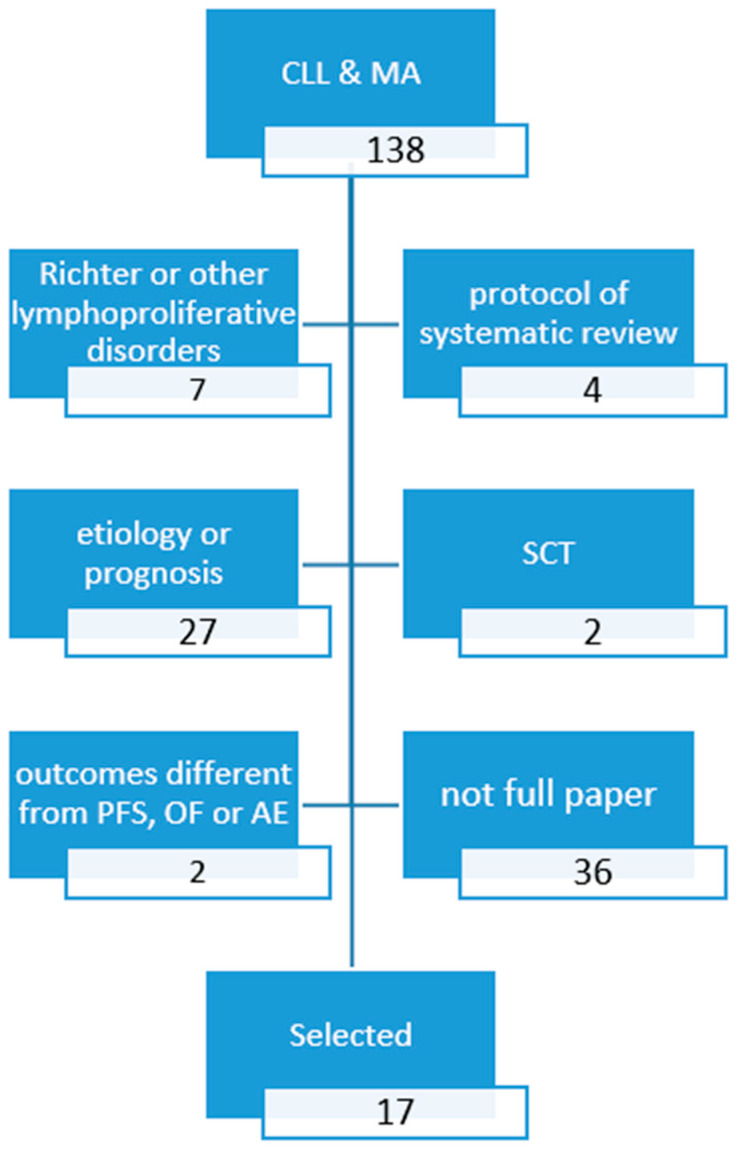
PRISMA diagram search a): Fully published (article or review) meta-analyses since 2016.

We searched Embase (Excerpta Medica database), the largest bibliographic database, selecting MAs and NMAs published in the last 5 years comparing pharmacologic therapies for CLL. The following query was used: ‘Leukemia, Lymphocytic, Chronic, B-Cell’/exp AND (eta-analysis)/lim AND (2016–2021)/py AND (English)/lim. A PRISMA (Preferred Reported Items for Systematic Reviews and Meta Analyses) diagram of the literature retrieval and selection process is shown in Figure 1. The enquiry was performed on 25 September 2021. The major results of the selected studies were reported according to the standard patient intervention comparison outcomes (PICO) format. We referred to the “recent standards of care” when ibrutinib (single-agent or associated with rituximab) or obinutuzumab–chlorambucil were the comparators in naïve patients. We referred to the “old standards of care” when bendamustine/rituximab, fludarabine-based therapies, or chlorambucil—as a single agent or in association with rituximab—were proposed as comparators in naïve patients.

From the selected studies, we retrieved data regarding OS, progression-free survival (PFS) and safety. More specifically, hazard ratios (HR) or risk ratios (RR), along with confidence or credible intervals (CI), were extracted.

## 3. Results

Overall, 138 records were retrieved: 13 fully published MAs reported PFS or OS (Table 1 and Table 2) [7,8,9,10,11,12,13,14,15,16,17,18,19] and 10 MAs reported safety outcomes (Table 3) [8,9,11,15,18,19,20,21,22,23].

### 3.1. Relative Survival Benefits Associated with Novel Drugs in Naïve CLL Patients

Four MAs compared frontline BTKis with both the older and recent standards of care (Table 1). A sponsored NMA compared ibrutinib with nine treatments based on alkylating agents and reported the PFS HRs to be 0.16 (0.08–0.31) when compared with chlorambucil, 0.82 (0.35–1.88) when compared with obinutuzumab–chlorambucil, and 0.72 (0.32–1.61) when compared with fludarabine–cyclophosphamide–rituximab (FCR) [11]. A non-sponsored MA confirmed a favorable PFS of ibrutinib-based frontline therapies in IGVH-mutated and unmutated naïve patients, as well as in those carrying the 11q deletion [12]. A significant OS advantage was not demonstrated by the above study [12]; however, an NMA that included 15 studies reported superior OS with ibrutinib compared to single-agent alkylating agents (FCR) and the combination of chlorambucil with ofatumumab [11]. 

Three NMAs [7,8,9] assessed the comparative survival of acalabrutinib-based therapies versus several other treatment options; however, only one NMA [7] used current standard treatments among the comparators. As a result, acalabrutinib had a superior PFS, but not OS, when compared to obinutuzumab–chlorambucil (HR 0.26; 95% CI 0.10–0.27) and heterogeneous PFS HRs when compared to ibrutinib or ibrutinib–rituximab. In particular, PFS was not significantly ameliorated by acalabrutinib as compared to ibrutinib when cross-trial comparisons were included in the NMA. 

Finally, three NMAs cross-compared the most recently proposed frontline therapies, namely acalabrutinib, acalabrutinib–obinutuzumab, ibrutinib–obinutuzumab, and venetoclax–obinutuzumab, following three randomized trials (ILLUMINATE, ELEVATE-TN, and CLL-14) [8,9,13]. The single agent acalabrutinib did not appear to significantly prolong PFS when compared to the obinutuzumab-based combinations with ibrutinib or venetoclax, while indirect comparisons consistently showed a superior expected PFS of acalabrutinib–obinutuzumab against obinutuzumab–venetoclax and obinutuzumab–ibrutinib. The acalabrutinib-based therapies showed a significant OS advantage in indirect comparison with chlorambucil and its combinations with rituximab or ofatumumab. However, the acalabrutinib-based therapies did not show an OS superior to that of the current standard treatments, namely ibrutinib or obinutuzumab–chlorambucil.

### 3.2. Relative Survival Benefits Associated with Novel Drugs in Refractory/Relapsed CLL Patients

Six MAs were devoted to R/R patients (Table 2); however, only two of them [17,18] compared BTK inhibitors to mixed comparators. The others either adopted the single agent ofatumumab as a comparator, which is a rarely used treatment, or addressed maintenance therapies, which are not reimbursed in most countries. Both PFS and OS were significantly enhanced by BTK inhibitors: the HR was 0.24 (0.19–0.30) for PFS and 0.58 (0.46–0.73) for OS. Rituximab–venetoclax was compared to rituximab–bendamustine or ofatumumab monotherapy in two MAs [14,16], which reported a considerable amelioration of both PFS and OS. In addition, the combinations of BTK inhibitors with rituximab and bendamustine were indirectly compared with rituximab–bendamustine and OS HRs of 0.20 (0.5–0.28) and 0.33 (0.25–0.44) were reported for ibrutinib-based and idelalisib-based combinations, respectively. 

### 3.3. Safety of Novel Drugs in CLL Patients

Ten MAs analyzed the safety outcomes of different pharmacologic therapies for CLL patients (Table 3). Four MAs compared BTK inhibitors to mixed standards of care that did not include BTK inhibitors or BCL2 inhibitors; discontinuation due to adverse events (AE) was not significantly different. However, a considerable increased risk of infections (OR 1.24; 1.02–1.50) was reported in relapsed/refractory (R/R) patients. Conversely, a much lower risk of discontinuation was associated with ibrutinib in naïve patients that were not eligible for fludarabine therapy. The above MAs reported that in mixed populations including naïve and R/R patients, abdominal AE, arthralgia, any-grade bleeding, arterial hypertension, and atrial fibrillation, were higher with ibrutinib treatment, as compared to alkylating agents. Similarly, in two MAs with R/R CLL patients, besides a small portion of lymphomas, high-grade adverse events were higher and the risk ratio for major bleedings was 2.46 (1.37–4.41) in patients receiving ibrutinib. 

No significantly lower risk of adverse events was reported when patients were given acalabrutinib or its combination with obinutuzumab versus obinutuzumab–venetoclax or obinutuzumab–ibrutinib.

### 3.4. Partially Reported Meta-Analyses

An additional search was performed to analyze MAs or NMAs reported at international meetings in the last two years that were not accessible as full texts. We retrieved six relevant NMAs [24,25,26,27,28,29]. Most of these meta-analyses addressed safety outcomes. In particular, the meta-analyses confirmed an increased risk of cardiovascular adverse events (a 3.7-fold increase) and ventricular arrhythmias (relative risk 8.13; 4.37–15.10) in patients treated with ibrutinib [24,27]. However, in VigiBase^®^, the relative risk of reporting (ROR) of cardiovascular adverse events was also higher in patients treated with venetoclax and idelalisib, and, more specifically, it was similarly high in patients treated with ibrutinib (ROR 3.06; 2.81–3.21) and acalabrutinib (ROR 2.66; 1.27–5.58) [24]. A more recent NMA that included 27 trials estimated a significant difference favoring acalabrutinib versus ibrutinib for arterial hypertension, atrial fibrillation, grade 3 arterial hypertension (OR 0.15, 95% CI 0.08–0.27; *p* < 0.0001), and grade 3 atrial fibrillation (OR 0.04, 95% CI 0.01–0.25; *p* = 0.0009) [25]. A meta-regression analysis indicated that the incidence of ventricular arrhythmias associated with ibrutinib exposure increased with a longer duration of treatment (coefficient = 0.0206, *p* = 0.049) and age (greater than 60 years; coefficient = 0.0237, *p* = 0.044) [27].

Non-cardiovascular adverse events related to ibrutinib were also analyzed [25]. Significant differences were reported in AE rates including headache (12% vs. 37%) and infections (35% vs. 57%) favoring ibrutinib versus acalabrutinib. However, an indirect NMA comparison revealed that grade 3 infections were significantly less frequent in patients treated with acalabrutinib (OR 0.62, 0.46–0.85; *p* = 0.003), after adjusting for age and median follow-up. Additionally, cytopenias and myalgias were reported less frequently in patients treated with acalabrutinib. Bleedings were not reported to be significantly higher in patients treated with ibrutinib versus acalabrutinib [25].

Three NMAs addressed survival outcomes by comparing obinutuzumab–acalabrutinib with other obinutuzumab-based therapies [26] and obinutuzumab–venetoclax with other therapies [26,29]. By including seven and ten studies enrolling naïve CLL patients, respectively, two NMAs reported a superior outcome of obinutuzumab–venetoclax when compared to rituximab-based chemotherapy, and also rituximab–ibrutinib in unfit patients [29]. However, an indirect comparison in naïve fit CLL patients between obinutuzumab–venetoclax and obinutuzumab–ibrutinib did not show significantly different PFS [29].

## 4. Discussion

Both life expectancy and quality of life in patients with B-cell chronic lymphocytic leukemia (CLL) are progressively improving owing to an increasing number of safer and more effective pharmacologic therapies. However, the speed of new drug introduction has resulted in differences in the standard of care and compromised robust technology assessments. Moreover, as the incidence of CLL is higher than 5 in 10,000 people, high-cost therapies for CLL may pose significant budget hurdles for the healthcare system.

This review aimed to systematically retrieve a comparative evidence base for the approval and adoption of novel CLL drugs. NMAs allow for an indirect comparison of treatments that are not directly compared in clinical trials, owing to Bayesian networks. Despite their limitations, NMAs have become standard tools, which have also been approved by regulatory agencies, for comparing new treatments with composite comparators. Therefore, we conducted an umbrella analysis by analyzing MAs and NMAs published since 2016 that addressed biologic therapies for CLL, and retrieved 20 meta-analyses that were fully (17) or partially (three) published. We did not take into account those studies addressing intermediate outcomes, such as clinical or molecular responses [30] or cellular therapies [31]. Several of the retrieved MAs included a large list of comparators, ranging from the single-agent chlorambucil to venetoclax- or ibrutinib-based therapies. The information mined from the MAs, however, allowed us to highlight some consistent associations. In particular, acalabrutinib as a single agent and obinutuzumab in combination with BTK or BCL-2 inhibitors demonstrated significantly longer PFS than ibrutinib, ibrutinib–rituximab, and obinutuzumab–chlorambucil, which are the current standard of treatment for naïve patients. However, a significantly superior OS of the above frontline treatments was reported by uniquely comparing acalabrutinib-based therapies with chemotherapy not including obinutuzumab. 

Our umbrella analysis also addressed the safety of drugs. Treatment with BTK inhibitors, namely ibrutinib, reported significantly higher rates of arterial hypertension, atrial fibrillation, major bleedings, and arthralgia than non-BTK-based therapies. In only one partially reported NMA, acalabrutinib showed better cardiovascular and infective outcomes than ibrutinib, but similar bleeding events.

Some limitations of the present umbrella analysis should be highlighted. Firstly, several retrospective comparisons among treatments for CLL were excluded from almost all the retrieved MAs. However, real-life studies are of paramount importance, since they also include a large portion of patients who are usually excluded from clinical trials because of comorbidities or concurrent neoplasms [32,33]. Moreover, very rare adverse events, such as immune cytopenias, can rarely be tracked by randomized studies [34]. Another important limitation is that many of the retrieved MAs were sponsored by pharmaceutical companies and company employees were included among the authors. This is a major conflict of interest which may have partially biased the results and should be carefully considered. 

In conclusion, novel combination therapies for CLL consistently prolong PFS when compared to the current standard of care, while OS is improved only in comparison with old therapies. The safety advantages of acalabrutinib-based therapies still need to be fully exploited in naïve patients, compared to the current standard of care; however, they are expected to be favorable. Based on the present umbrella analysis, we can remind scientific societies elaborating clinical practice guidelines to highlight critical outcomes for each recommendation, according to the current standards for evidence-based evidence [35,36]. Furthermore, we expect that regulatory agencies agree with the stakeholders that comparators and major endpoints are valuable for approving new drugs for CLL. This is a heated debate since future therapies for pluri-relapsed and multi-refractory “difficult-to-treat” CLL patients include powerful cellular therapies, such as autologous CAR-T therapy. A recent systematic review of nine published studies (208 patients overall) reported cytokine release syndrome in 15% and neurotoxicity in 13% of the treated patients; however, a complete response was achieved in 40% of the treated patients [31]. While survival data are being collected, the appropriate threshold risk/benefit and cost/benefit ratios of innovative therapies for CLL should be further investigated [37,38,39].

## Figures and Tables

**Table 1:** Fully published MAs and NMAs since 2016: PFS and OS in naïve patients [7,8,9,10,11,12,13].

Author, Year	NMA	Sponsored	N Studies (Patients)	Intervention	Comparator	Hazard Ratio or Risk Ratio (Confidence or Credible Intervals)
						**PFS**
Stadler, 2016 [10]	Yes	Yes	5 (2882)	O-Chl	Chl F Ofa-Chl R-Chl RB FCR-lite	**0.19 (0.14–0.25)** **0.20 (0.13–0.49)** **0.33 (0.23–0.49)** **0.43 (0.34–0.54)** 0.81 (0.49–1.33) 0.88 (0.51–1.52)
Xu, 2018 [11]	Yes	Yes	15 (5300)	I	Chl Flud O-Chl FC FCR B R-Chl Ofa-Chl RB	**0.16 (0.08, 0.31)** **0.19 (0.09, 0.38)** 0.82 (0.35, 1.88) **0.38 (0.18, 0.78)** 0.72 (0.32, 1.61) 0.71 (0.31, 1.65) **0.33 (0.15, 0.71)** **0.28 (0.13, 0.63)** 0.55 (0.24, 1.28)
Sheng, 2020 [9]	Yes	No	3 (1017)	OA	OI OV	**0.43 (0.22–0.87) 0.46 (0.22–0.96)** § **0.30 (0.15–0.59) 0.34 (0.17–0.68)** §
Davids, 2020 [7]	Yes	Yes	8 (3778)	A	I OI IR OV BR Chl O-Chl Ofa-Chl R-Chl	**0.35 (0.18–0.66)** 0.61 (0.32–1.15) ^ 0.87 (0.46–1.63) 0.63 (0.32–1.27) ^ **0.37 (0.18–0.75)** 0.56 (0.27–1.14) ^ 0.60 (0.33–1.11) **0.47 (0.24–0.89) ^** **0.15 (0.08–0.27) 0.19 (0.10–0.35)** **^** **0.04 (0.02–0.07) 0.03 (0.02–0.06)** **^** **0.20 (0.13–0.31) 0.16 (0.10–0.27)** **^** **0.07 (0.04–0.12) 0.06 (0.03–0.10)** **^** **0.08 (0.05–0.14) 0.07 (0.04–0.13)** **^**
				OA	I OI IR OV BR Chl O-Chl Ofa-Chl R-Chl	**0.19 (0.09–0.38) 0.46 (0.23–0.92)^** **0.46 (0.23–0.94)** 0.48 (0.23–1.01) ^ **0.20 (0.09–0.44) 0.43 (0.20–0.91)** **^** **0.32 (0.16–0.64) 0.36 (0.18–0.71)** **^** **0.08 (0.04–0.16) 0.14 (0.07–0.28)** **^** **0.02 (0.01–0.04) 0.02 (0.01–0.05)** **^** **0.11 (0.06–0.18) 0.12 (0.07–0.22)** **^** **0.04 (0.02–0.07) 0.04 (0.02–0.08)** **^** **0.04 (0.02–0.08) 0.06 (0.03–0.10)** **^**
Molica, 2020 CLM [8]	Yes	No	3 ^1^ (1191)	A	OI OV	0.87 (0.47–1.61) 0.57 (0.32v1.03)
				OA	OI OV	**0.43** **(0.22–0.87)** **0.29** **(0.15–0.56)**
				OV	OI	1.52 (0.82–1.81)
Molica, 2020 EJH [12]	No	No	4 (1574)	I +/− R/O	Mixed chemo (Chl, O-Chl, RB, FCR)	**0.331 (0.272–0.403)** **0.159 (0.077–0.327)** 11q- **0.178 (0.121–0.261)** IGVH unmut **0.270 (0.149–0.489)** IGVH mut
Chatterjee, 2021 [13]	Yes	Yes	6	A OA BR OI I IR	OV	0.6 (0.3–1.0) **0.4** **(0.2–0.8)** **6.9** **(3.3–13.2)** 0.9 (0.5–1.6) **2.5** **(1.4–4.3)** **2.8** **(1.2–5.4)**
						**OS**
Stadler, 2016 [10]	Yes	Yes	5 (2882)	O-Chl	F Chl Ofa-Chl R-Chl RB	0.35 (0.07–1.86) **0.48** **(0.30–0.78)** 0.53 (0.28–1.04) 0.81 (0.52–1.26) 0.81 (0.37–1.76)
Xu, 2018 [11]	Yes	Yes	15 (5300)	I	Chl Flud O-Chl FC FCR B R-Chl Ofa-Chl RB	**0.16(0.04, 0.56)** **0.15(0.04, 0.53)** 0.41 (0.09, 1.70) **0.14(0.04, 0.52)** **0.20(0.05, 0.79)** **0.21(0.05, 0.80)** 0.27 (0.06, 1.05) **0.18(0.04, 0.71)** 0.30 (0.06, 1.29)
Sheng, 2020 [9]	Yes	No	3 (1017)	OA	OI OV	0.51 (0.18–1.44) 0.38 (0.13–1.08)
Davids, 2020 [7]	Yes	Yes	8 (3778)	A	I OI IR OV BR Chl O-Chl Ofa-Chl R-Chl	0.44 (0.16–1.27) 0.66 (0.25–1.75) ^ 0.65 (0.24–1.75) 0.45 (0.15–1.40) 0.64 (0.22–1.87) ^ 0.48 (0.18–1.30) 0.45 (0.16–1.27) 0.61 (0.23–1.60) ^ **0.23** **(0.09–0.59)** **0.27 (0.11–0.70) ^** 0.60 (0.28–1.26) 0.59 (0.28–1.26) ^ **0.25 (0.09–0.71)** **0.30 (0.11v0.85) ^** **0.38 (0.15–0.94)** 0.44 (0.18–1.07) ^
				AO	I OI IR OV BR Chl O-Chl Ofa-Chl R-Chl	0.35 (0.12–1.04) 0.53 (0.19–1.45) ^ 0.51 (0.18–1.45) 0.36 (0.11–1.15) 0.51 (0.17–1.54) ^ 0.38 (0.13–1.08) 0.36 (0.12–1.05) 0.48 (0.17–1.34) ^ **0.18 (0.07–0.48)** **0.22 (0.08–0.58) ^** 0.47 (0.21–1.06) **0.20 (0.07–0.59)** **0.24 (0.08–0.71) ^** **0.30 (0.12–0.78)** **0.35 (0.14–0.88) ^**
Molica, 2020 EJH [12]	No	No	3 (1027)	I +/− R I +/− O	Mixed chemo +/− R/O	0.289 (0.07–1.175)
Chatterjee, 2021 [13]	Yes	Yes	6	A OA BR OI I IR	OV	0.6 (0.3–1.2) 0.5 (0.1–1.1) 1.2 (0.5–2.4) 1.0 (0.4–2.1) 1.2 (0.5–2.3) 1.2 (0.4–2.6)

Bolded hazard ratios show significantly reduced hazards of death of intervention versus comparator. ^§^ as by investigators’ assessment. ^1^ ILLUMINATE, ELEVATE-TN, and CLL14. ^ results of network B including cross-trial comparisons. & includes lymphomas different from CLL. Legend: A—acalabrutinib; BR—bendamustine plus rituximab; Chl—chlorambucil; F—fludarabine; FC—fludarabine and cyclo; A—acalabrutinib; BR—bendamustine plus rituximab; Chl—chlorambucil; F—fludarabine; FC—fludarabine and cyclophosphamide; FCR—fludarabine plus cyclophosphamide plus rituximab; FI—fludarabine ineligible; HG—grade 3–5; HR—hazard ratio; I—ibrutinib; IGVH—immunoglobulin heavy chain variable region; IR—ibrutinib plus rituximab; maint—maintenance; mut—mutated status; O—obinutuzumab; O-Chl—obinutuzumab plus chlorambucil; OA—obinutuzumab plus acalabrutinib; Ofa—ofatumumab; Ofa-Chl—ofatumumab plus chlorambucil; OI—obinutuzumab plus ibrutinib; OS—overall survival; OV—obinutuzumab plus venetoclax; PFS—progression-free survival; R—rituximab; R-Chl—chlorambucil plus rituximab; R/R—relapsed/refractory; RB—rituximab plus bendamustine; RV—rituximab plus venetoclax; V—venetoclax; unmut—unmutated status.

**Table 2:** Fully published MAs and NMAs since 2016: PFS and OS in relapsed/refractory (R/R) patients [14,15,16,17,18,19].

Author, Year	NMA	Sponsored	N Studies (Patients)	Intervention	Comparator	Hazard Ratio or Risk Ratio (Confidence or Credible Intervals)
						**PFS**
Wu, 2017 [19]	No	No	13 (2314)	Ofa-based	Non-Ofa-based	0.88 (0.47–1.63)
Pula, 2018 [18]	No	No	5 (1866)	BTK inhibitors	Non-BTK inhibitors	****0.24** **(0.19–0.30)****
Chen, 2019 [14]	Yes	No	7 (2514)	RV I	Ofa	**0.10** **(0.05–0.21)** **0.10** **(0.07–0.17)**
Lee, 2020 [15]	No	Yes	6 (1615)	Lenalidomide (maint) R (maint) Ofa (maint)	No maintenance	**0.37** **(0.27–0.50)** **0.50** **(0.38–0.66)** **0.52** **(0.41–0.66)**
Molica, 2019 [17]	No	No	7 (2409)	I or A or V +/− R/O	No BTK inhibitor nor venetoclax	**0.187 (0.126–0.279)** non 17p- **0.240 (0.185–0.311)** TP53wt **0.239 (0.166–0.344)** IGVH mutated **0.208 (0.168–0.59)** non 11q- **0.206 (0.108–0.392)** 17p- **0.231 (0.137–0.390)** TP53 mutated **0.172 (0.109–0.272)** IGVH unmutated **0.081 (0.054–0.121)** 11q-
						**OS**
Wu, 2017 [19]	No	No	13 (2314)	Ofa-based	Non-Ofa-based	0.97 (0.70–1.36)
Pula, 2018 [18]	No	No	5 (1866)	BCR-inhibitors	Non BCR-inhibitors	**0.58** **(0.46–0.73)**
Chen, 2019 [14]	Yes	No	7 (2514)	RV I	Ofa	**0.33** **(0.11–0.99)** **0.36** **(0.21–0.63)**
Molica, 2020 LL [16]	Yes	No	3 (1383)	RV RB + I RB + idelalisib	RB	**0.17** **(0.11–0.25)** **0.20** **(0.15–0.28)** **0.33** **(0.25–0.44)**
Lee, 2020 [15]	No	Yes	6 (1615)	Lenalidomide, R, or Ofa maintenance	No maintenance	0.89 (0.70–1.14)

Bolded risk ratios show significantly increased (or reduced) hazards of adverse events of intervention versus comparator. Legend: A—acalabrutinib; BR—bendamustine plus rituximab; Chl—chlorambucil; F—fludarabine; FC—fludarabine and cyclo; A—acalabrutinib; BR—bendamustine plus rituximab; Chl—chlorambucil; F—fludarabine; FC—fludarabine and cyclophosphamide; FCR—fludarabine plus cyclophosphamide plus rituximab; FI—fludarabine ineligible; HG—grade 3–5; HR—hazard ratio; I—ibrutinib; IGVH—immunoglobulin heavy chain variable region; IR—ibrutinib plus rituximab; maint—maintenance; mut—mutated status; O—obinutuzumab; O-Chl—obinutuzumab plus chlorambucil; OA—obinutuzumab plus acalabrutinib; Ofa = ofatumumab; Ofa-Chl—ofatumumab plus chlorambucil; OI—obinutuzumab plus ibrutinib; OS—overall survival; OV—obinutuzumab plus venetoclax; PFS—progression-free survival; R—rituximab; R-Chl—chlorambucil plus rituximab; R/R—relapsed/refractory; RB—rituximab plus bendamustine; RV—rituximab plus venetoclax; V—venetoclax; unmut—unmutated status.

**Table 3:** Fully published MAs and NMAs since 2016: safety outcomes [8,9,11,15,18,19,20,21,22,23].

Author, Year	NMA	Sponsored	N Studies (Patients)	Population	Intervention	Comparator	Outcome	Hazard Ratio or Risk Ratio (Confidence or Credible Intervals)
Wu, 2017 [19]	No	No	13 (2314)	R/R	Ofa-based	Non-Ofa-based	AE	Infections more frequent Thrombocytopenia & anemia less frequent
Pula, 2018 [18]	No	No	5 (1866)	R/R	BTK inhibitors	Non BTK inhibitors	AE HG AE disc AE death	**1.25 (1.08–1.44)** 1.26 (0.88–1.81) 1.06 (0.72–1.57)
Xu, 2018 [11]	Yes	Yes	15 (5300)	Naïve	I	Chl O-Chl R-Chl Ofa-Chl B	AE disc	0.32 (0.08–1.18) 0.31 (0.05–2.00) 0.66 (0.1–4.31) 0.31 (0.05–1.90) **0.08** **(0.1–0.6)**
				Naïve, FI	I	Chl O-Chl R-Chl Ofa-Chl B	AE disc	**0.23(0.15–0.63)** **0.31(0.11–0.80)** 0.65 (0.23–1.81) **0.31(0.12–0.77)** **0.08(0.02–0.7)**
Zhou 2019 [21]	No	No	5 (2456)	Naïve, R/R	I	Mixed	Anemia Thrombocytopenia Neutropenia Febrile neutropenia Respiratory tract infections Abdominal AE Arthralgia	0.90 (0.67–1.21) 0.61 (0.32–1.14) 0.50 (0.25–1.00) 0.89 (0.32–2.49) 1.01 (0.78–1.30) **2.14** **(1.44–3.17)** **1.86** **(1.10–3.15)**
Caldeira, 2019 [23]	No	No	8 (2580)	CLL &	I-based therapy	Mixed	Arterial hypertension Atrial fibrillation	**2.82** **(1.52–5.23)** **4.69** **(2.17–7.64)**
Wang 2020 [22]			11 (4288)	CLL &	I	Mixed	Bleeding Major bleed	**3.08** **(2.07–4.58)** **2.46** **(1.37–4.41)**
Ball, 2020 [20]	No	No	5 (1739)	Naïve, R/R	I	Mixed	Infections HG	**1.24** **(1.02–1.50)**
Lee, 2020 [15]	No	Yes	6 (1615)	R/R	Lenalidomide (maint) R (maint) Ofa (maint)	No maintenance	AE	1.84 (0.98–3.43) 1.11 (0.69–1.79) 2.11 (0.92–4.81)
Sheng, 2020 [9]	Yes	No	3 (1017)	Naïve	OA	OI	AE disc Grade 3–4 AE Any AE	0.64 (0.11–1.86) 1.10 (0.52–2.32) 0.48 (0.01–48.20)
					OA	OV	AE disc Grade 3–4 AE Any AE	0.68 (0.26–1.81) 5.28 (0.03–831.44) 0.89 (0.45–1.77)
Molica, 2020 CLM [8]	Yes	No	3 (1027)	Naïve	VO A A	IO IO VO	Grade 3–4 AE	1.05 (0.64–1.73) 0.73 (0.43–1.24) 0.69 (0.44–1.09)

Bolded risk ratios show significantly increased (or reduced) hazards of adverse events of intervention versus comparator. Legend: A—acalabrutinib; AE—adverse effects; AE disc—discontinuation for an AE; BR—bendamustine plus rituximab; Chl—chlorambucil; F—fludarabine; FC—fludarabine and cyclo; A—acalabrutinib; BR—bendamustine plus rituximab; Chl—chlorambucil; F—fludarabine; FC—fludarabine and cyclophosphamide; FCR—fludarabine plus cyclophosphamide plus rituximab; FI—fludarabine ineligible; HG—grade 3–5; HR—hazard ratio; I—ibrutinib; IGV—immunoglobulin heavy chain variable region; IR—ibrutinib plus rituximab; maint—maintenance; mut—mutated status; O—obinutuzumab; O-Chl—obinutuzumab plus chlorambucil; OA—obinutuzumab plus acalabrutinib; Ofa—ofatumumab; Ofa-Chl—ofatumumab plus chlorambucil; OI—obinutuzumab plus ibrutinib; OS—overall survival; OV—obinutuzumab plus venetoclax; PFS—progression-free survival; R—rituximab; R-Chl—chlorambucil plus rituximab; R/R—relapsed/refractory; RB—rituximab plus bendamustine; RV—rituximab plus venetoclax; V—venetoclax; unmut—unmutated status.
